# Survival After Hip Fracture: A Comparative Analysis Between a Private and a Public Health Center in Chile

**DOI:** 10.7759/cureus.11773

**Published:** 2020-11-29

**Authors:** Maximiliano Barahona, Alvaro Martinez, Cristian Barrientos, Macarena A Barahona, Gabriel Cavada, Julian Brañes

**Affiliations:** 1 Orthopaedic Department, Hospital Clinico Universidad De Chile, Santiago, CHL; 2 Orthopaedic Department, Hospital San Jose, Santiago, CHL; 3 Orthopaedic Department, Hospital Clinico Universidad de Chile, Santiago, CHL; 4 Orthopaedic Department, Clinica Santa Maria, Santiago, CHL; 5 Epidemiology Department, Universidad de Chile, Santiago, CHL

**Keywords:** hip fractures, mortality rate, survival, epidemiology, health inequity

## Abstract

Purpose

The purpose of the study is to compare the survival after hip fracture in patients older than 50 years after hip fracture between a private and a public health center in Chile. We hypothesize that treatment at a private health center (PRH) may be associated with lower one-year mortality and longer median survival time after hip fracture (adjusted by gender and age) compared to a public health center (PLH).

Methods

PRH and PLH patients who were coded with a diagnosis of hip fracture were included in this study. PRH patients were included between 2002 to 2018, and PLH patients were included from 2012 to 2018. One-year mortality was estimated by logistic regression; meanwhile, median survival time was estimated by exponential regression. A survival analysis study was designed and approved by our institutional ethics review board.

Results

A total of 2130 patients were included in the PLH cohort, and a total of 1110 patients were included in the PRH. The one-year mean mortality, adjusted by age and gender, was 0.23 (range: 0.21 to 0.25) in the PLH and 0.16 (range: 0.13 to 0.18) in the PRH cohort. The median survival time, adjusted by age and gender, was 4.2 years (range: 4.1 to 4.4) in the PLH and 6.8 years (range: 6.3 to 7.29) in the PRH cohort.

Conclusion

Patients older than 50 years treated in a private health center have a higher median survival time and a lower probability of dying one year after a hip fracture.

## Introduction

Hip fracture is a frequent pathology in patients aged ≥ 50 years. Therefore, an upward trend in the incidence of hip fracture is expected due to the overall aging of the population [1]. The gold standard for its treatment is to undergo surgery as soon as possible after hospital admission [2]. The one-year mortality rate after a hip fracture has been classically described as one in three cases. Recent reports show a significant downward trend in one-year mortality rates worldwide, reaching 23.3% in Europe, 17.9% in Asia, and 24.9% in the United States [3]. In addition, studies show lower survival in patients with osteoporotic bone fracture than in the general population, so only considering the one-year mortality in hip fracture cases seems biased [4].

Relatively fast economy growth in Chile in the last three decades has achieved a significant reduction in absolute poverty, but Chile remains a country with one of the more unequal distributions of wealth among citizens and residents, reaching a Gini coefficient of 0.46 in 2017 [5, 6]. The Chilean health system relies heavily on private health centers (PRHs) and out-of-pocket health expenditures for its residents, despite 73.5% of the population belonging to the national health fund (i.e., the public health network) [7]. Inequity is a prominent issue in Chile, and timely and quality access to health care are not excluded from the problem [8]. Chile PRHs rely on a performance-based compensation for healthcare professionals (where they are paid per surgery or patient case), while the public health centers (PLH) pay based on a fixed amount regardless of the number of surgical procedures performed during a scheduled work shift. The former payment method is associated with significantly lower one-year mortality after hip fracture [9].

One national study performed in the V region of Chile from 2010 to 2012 included a cohort of patients aged >60 years with a hip fracture treated in a PLH. A total of 617 patients with a mean age of 81 years were included. The authors found a one-year mortality rate of 0.27 [10]. To our knowledge, no other epidemiologic study of hip fractures in Chile has been published.

The purpose of this study is to perform a survival analysis of patients older than 50 years after hip fracture. We hypothesize that treatment in PRH is associated with lower one-year mortality and longer median survival time after hip fracture adjusted by gender and age than treatment in PLH due to shorter waiting time for surgery and a higher proportion of patients that undergo surgery. 

## Materials and methods

A survival analysis study was designed and approved by an institutional ethics review board. The patients were recruited from two health centers (one PRH, one PLH). Both centers are at a distance of 1.1 kilometers, according to Google Maps® (Alphabet, Inc) in Santiago, the capital city of Chile. Both centers have access to the same type and brands of the osteosynthesis material and prostheses to perform surgery, and surgeons have similar levels of training, and two surgeons work in both centers. Both health centers codify diseases according to the 10th International Classification of Diseases (ICD-10); a search was conducted for codes S72.0 (head and a neck fracture of the femur), S72.1 (pertrochanteric fracture), and S72.2 (subtrochanteric fracture of the femur). All those patients who were coded with a diagnosis of hip fracture were included in this study. In the PRH, the revision was performed between 2002 to 2018. In the PLH, the revision was conducted from 2012 to 2018. Unfortunately, data before 2012 were not available in the PLH. The patient’s full name, national identificatory number, birth date, date of admission, date of discharge, surgery code, and surgery date were recorded. Patients with incomplete information were excluded. Then, per the National Portal of Transparency, we requested the National Civil Registry of Chile to inform whether the patients were alive by July 30, 2019; if not, they were asked to report the date of death.

Continuous variables are summarized in median, range, and interquartile range (IQR, p25-p75), and absolute frequency and percent was used for discrete variables. The unmatched nonparametric median test was used to compare continuous variables, and Fisher’s exact test was used to compare discrete variables. A p-value of <0.05 was interpreted as significant.

Spearman’s correlation was used to evaluate the trend over the period studied for one-year mortality, waiting time for surgery, the proportion of patients that did not undergo surgery, and length of hospital stay. The rho statistic was reported. A positive rho indicated an upward trend, and negative rho reflected a downward trend. A p-value of <0.05 was interpreted as a significant correlation.

Multivariate logistic regression was estimated using one-year mortality as the dependent variable. Variables were included in the model if they reached a p<0.05. Pearson or Hosmer-Lemeshow goodness-of-fit test was used to validate the model. Post-estimation probabilities were calculated for each variable included in the model.

For survival analyses, only patients from 2012 to 2018 were included in the PRH to fit with the PLH period studied (2012-2018), as patients from more than five years from the target range could have a different life expectancy and may bias the results. The Kaplan-Meier curve was estimated by the center and compared with the log-rank test. To find the median survival time after hip fracture, two multivariate exponential regressions were estimated. The first model compared the survival time between surgery and non-surgery adjusted by age and gender. The second model compared the survival time between the waiting time for surgery using three groups: 0 to two days (<3), three to five days (3-5), and six days or more (>5). Both models were tested fitting the logarithm of the Kaplan-Meier product-limit estimate of the survivor function to the studied time and were accepted as it achieved a correlation of 0.99. A significance of 5% was used to include the independents variable into both models, and a confidence interval level of 95% was reported. The 2017 publication of the Statistics National Institute of Chile was used to compare this estimation to the expectancy of life of the general national population (GP) [11]. Also, assuming exponential distribution, the median survival time of the cohort of Dinamarca-Montecinos et al. was estimated [10]. Finally, the one-year mortality estimated by the logistic regression analysis was adjusted to the gender proportion and mean age reported by Dinamarca-Montecinos et al. [10], allowing for a comparison of the one-year mortality for the three cohorts. The data were processed using Stata version 15 (StataCorp LP, College Station, Texas, USA).

## Results

A total of 3240 patients were included; 2493 of them were women (76.94%). The median age was 82 years (range: 50 to 105; IQR: 74 to 87). The median hospital length of stay was nine days (range: 1 to 161; IQR: 6 to 14), and the median waiting time for surgery was five days (range: 1 to 92; IQR: 3 to 9). The one-year mortality rate was 0.21 (95% CI: 0.20 to 0.23), and the intrahospital mortality was 0.03 (95% CI: 0.01 to 0.05).

A total of 2130 patients were included in the PLH cohort, and a total of 1110 patients were included in the PRH cohort. The median age between groups was not statistically different, but a significantly higher proportion of women was found in the PRH. The median waiting time for surgery in the PLH was twice as long as the PRH, reaching a significant difference. The one-year mortality rate in the PRH is approximately two-thirds of the one-year mortality of the PLH, reaching a significant difference (Table 1).

**Table 1:** Comparison between PRH and PLH A comparison between PRH and PLH. Both are similar in age but significantly different in the other variables. n - absolute frequency; percentage - percentage frequency; m - median; iqr - interquartile range; CI - confidence interval; Ft - Fisher exact test; Mt - unmatched nonparametric median test

Variable	Private	Public	P
N	1110	2130	NA
Age (years) ^(m,iqr)^	82 (74–88)	82 (74–87)	0.189 ^Ft^
Female ^(n, percentage) ^	885 (79.7%)	1608 (75.5%)	0.006 ^Ft^
Hospital stay length (days) ^(m,iqr)^	8 (5–12)	10 (7–15)	<0.001 ^Mt^
No surgery ^(n, percentage)^	26 (2.21%)	209 (9.15%)	<0.001 ^Ft^
Wait time for surgery (days) ^(m,iqr)^	3 (2–5)	6 (4–11)	<0.001 ^Mt^
<3 days ^(n, percentage)^	401(37%)	465(24%)	
3–5 days ^(n, percentage)^	505 (47%)	354 (18%)	
>5 days ^(n, percentage)^	178 (16%)	1112 (58%)	<0.001 ^Ft^
One–year mortality ^(rate, 95% CI) ^	0.16 (0.14–0.19)	0.26 (0.24–0.27)	<0.001 ^Ft^
Intrahospital mortality ^(rate, 95% CI)^	0.009 (0.004–0.016)	0 (0–0.002)	<0.001 ^Ft^

In PLH, the hospital length of stay (Spearman -0.07, p=0.001), the waiting time for surgery (Spearman -0.12, p<0.000), and one-year mortality rate (Spearman -0.07, p=0.004) have a weak downward trend, but statistically significant. The PRH had no significant decrease in any of the mentioned variables, reaching a Spearman correlation of 0.02 (p=0.46), 0.03 (p=0.34), and -0.04 (p=0.18) for hospital length of stay, waiting time for surgery, and one-year mortality, respectively. Neither center had a significant downward trend in the proportion of patients that did not undergo surgery.

A total of 218 patients did not undergo surgery, of them 192 (88%) were treated in the public health network. The proportion of patients that did not receive surgery in PLH and PRH was 9.15% and 2.21%, respectively (Table 1). The global mortality at one-year in patients that did not undergo surgery was 0.60 (95% CI: 0.54 to 0.67), and 0.20 (95% CI: 0.18 to 0.21) in patients that underwent surgery. Patients that did not undergo surgery have a one-year mortality rate of 0.27 (95% CI: 0.12 to 0.48) in the PRH. Meanwhile, patients that did not undergo surgery in PLH reached one-year mortality of 0.65 (95% CI: 0.57 to 0.71). This difference reached statistical difference.

The multivariate logistic regression using one-year mortality as the dependent variable estimates an odds ratio (OR) of 1.05 (95% CI: 1.04 to 1.06) for every year, OR 1.88 (95% CI: 1.53 to 2.31) for men, OR 5.90 (95% CI: 4.37 to 7.97) for non-surgery and OR 1.60 (95% CI: 1.31 to 1.94) for PLH. This model estimates a one-year mortality rate, adjusted by age and gender in that patients underwent surgery, of 0.21 (95% CI: 0.19-0.23) in the PLH and 0.14 (95% CI: 0.12 to 0.16) in the PRH cohort. The model also estimates one-year mortality adjusted by age and gender in patients who did not undergo surgery of 0.61 (95% CI: 0.54 to 0.68) in a PLH and of 0.49 (95% CI: 0.41 to 0.58) in PRH. The total one-year mortality rate estimated is 0.16 (95% CI: 0.13 to 0.18) and 0.23 (95% CI: 0.21 to 0.25) in the PRH and PLH, respectively, adjusted by age and gender. The estimated probability of dying one year after hip fracture by year, gender, and center is summarized in Table 2.

**Table 2:** Probability of dying during the first year after hip fracture The probability and 95% confidence interval of dying during the first year after hip fracture by type of health center, group of age, gender, and access to surgery. PRH - private health center; PLH - public health center

Age (years), gender	PRH, no surgery	PLH, no surgery	PRH, surgery	PLH, surgery
50–59, F	0.19 (0.12–0.25)	0.27 (0.19–0.34)	0.04 (0.03–0.05)	0.06 (0.04–0.07)
50–59, M	0.30 (0.21–0.39)	0.41 (0.32–0.50)	0.07 (0.05–0.09)	0.10 (0.07–0.13)
60–69, F	0.28 (0.20–0.35)	0.38 (0.30–0.45)	0.06 (0.05–0.08)	0.09 (0.08–0.11)
60–69, M	0.42 (0.33–0.51)	0.53 (0.45–0.62)	0.11 (0.08–0.13)	0.16 (0.13–0.19)
70–79, F	0.40 (0.31–0.48)	0.51 (0.44–0.58)	0.10 (0.08–0.12)	0.15 (0.13–0.17)
70–79, M	0.55 (0.46–0.64)	0.66 (0.59–0.73)	0.17 (0.14–0.21)	0.25 (0.22–0.28)
>80, F	0.55 (0.47–0.63)	0.66 (0.59–0.73)	0.17 (0.15–0.20)	0.25 (0.23–0.28)
>80, M	0.70 (0.62–0.78)	0.79 (0.73–0.84)	0.28 (0.24–0.33)	0.38 (0.34–0.43)

**Figure 1:**
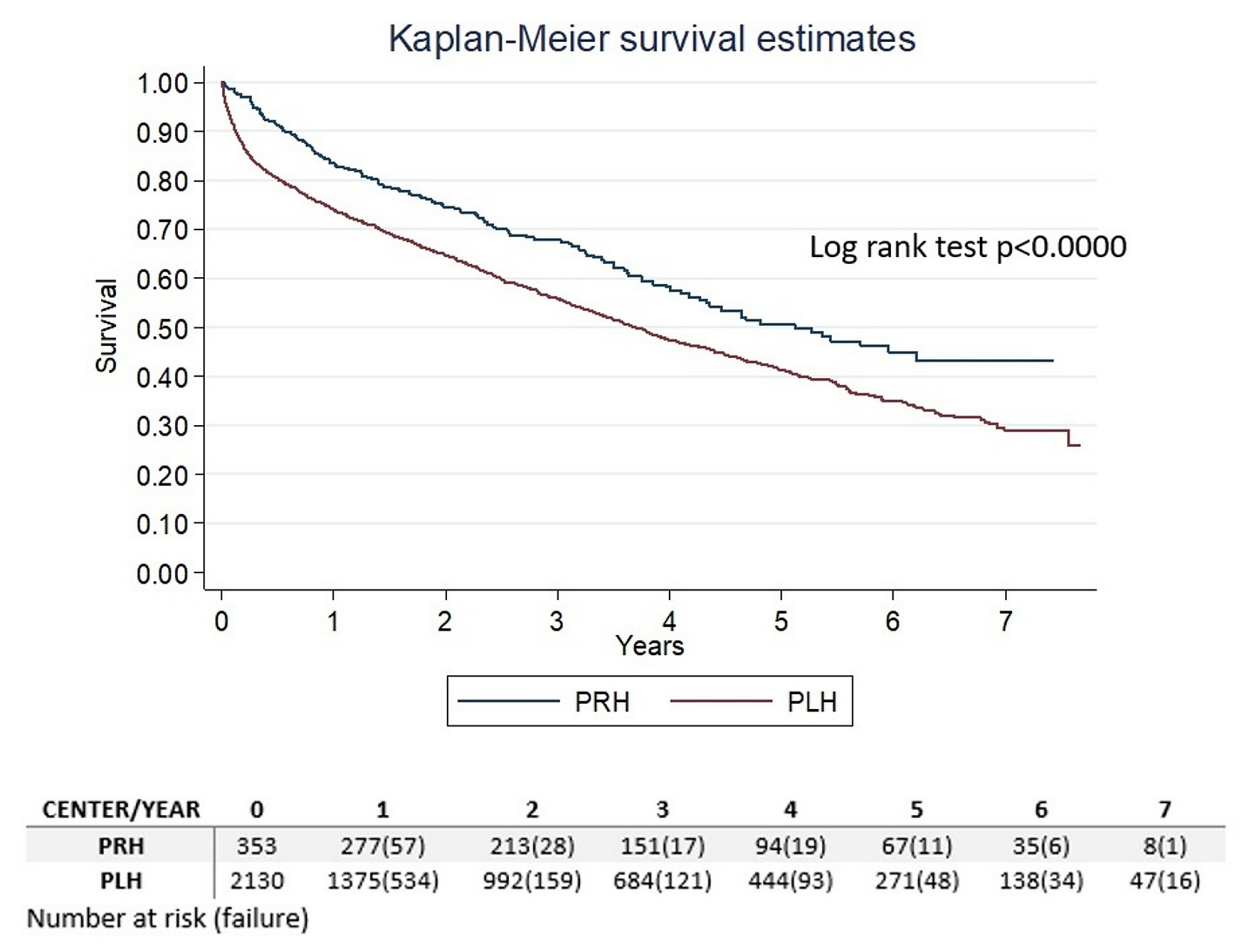
Kaplan-Meier estimate by center Kaplan-Meier estimate by center shows a significantly better survival rate for PRH (log-rank test p<0.0000). The number of patients at risk at every year are reported, also a failure at every time is shown between parenthesis. PRH - private health center; PLH - public health center

The total time at risk was 5890 years, the incidence rate of death was 0.19, the individual median time at risk was 1.9 years, and the median survival time was 3.8 years. Kaplan-Meier survival estimates, the number of patients at risk, and the number of failures at every year by center are shown in Figure 1.

**Figure 2:**
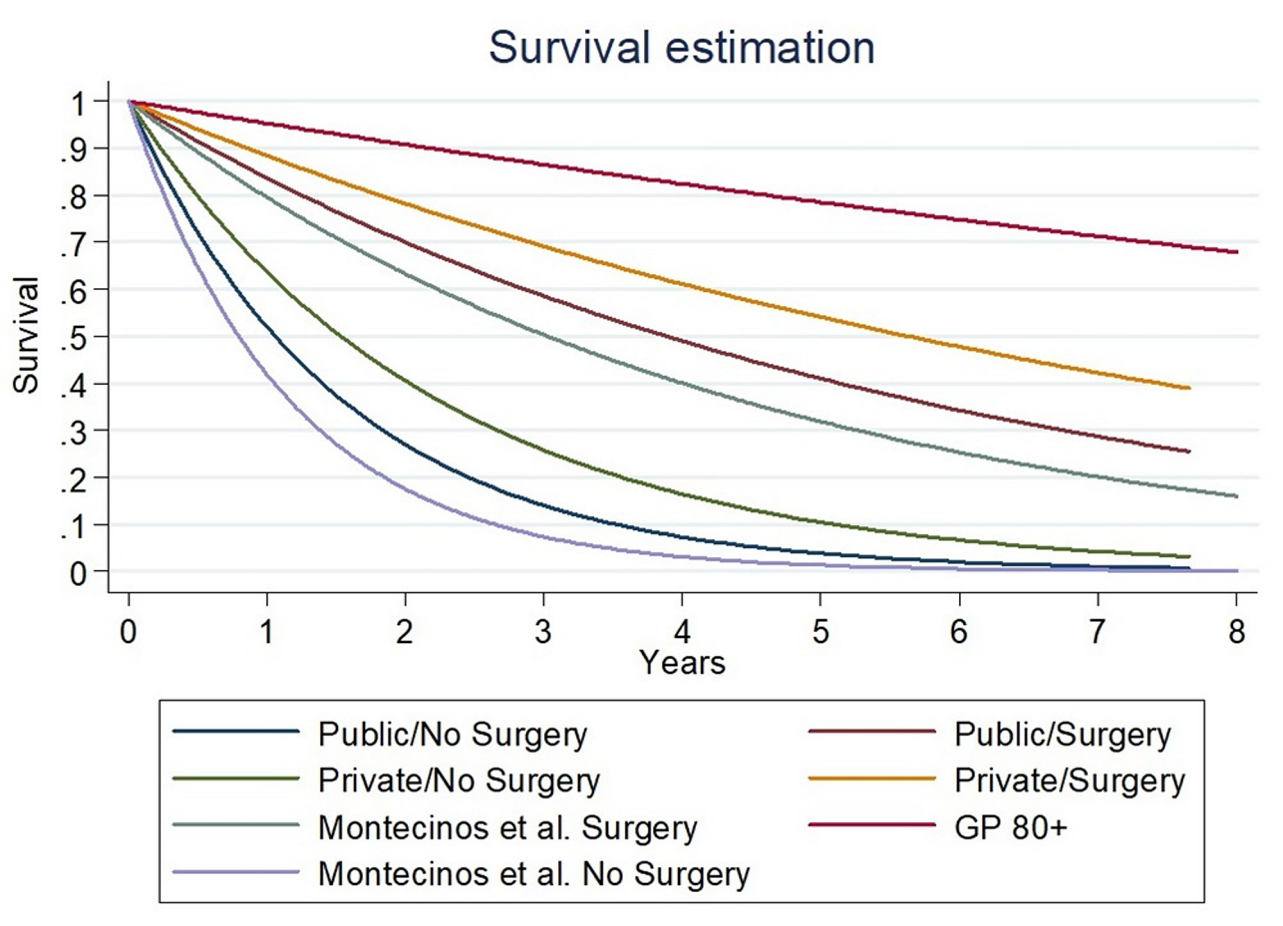
Survival curve estimated by exponential regression. Survival curve estimated by exponential regression. From best to worse: general population over 80 years old (GP 80+), private center and patient underwent surgery, public center and patient underwent surgery, Dinamarca-Montecinos et al. [10] cohort and patient underwent surgery, private center, and patient did not undergo surgery, private center and patient did not undergo surgery, Dinamarca-Montecinos et al. [10] cohort and patient did not undergo surgery.

Exponential regression estimates a hazard ratio (HR) of 1.05 (95% CI: 1.05 to 1.06) for each year of age, HR 1.61 (95% CI: 1.41 to 1.85) for men, HR 3.67 (95% CI: 3.09 to 4.37) for non-surgery, and HR 1.45 (95% CI: 1.21 to 1.73) for PLH. Both centers have shown a significantly lower survival rate after hip fracture compared to the general population. The estimated median survival time is summarized in Table 3. The survival curve estimated by the exponential regression model is shown in Figure 2.

**Table 3:** Life expectancy in years after hip fracture Life expectancy in years after hip fracture by type of health center, group of age, and access to surgery. Also, the general national population (GP) is shown in the second column, on the left, the male expectancy of life, and on the right, the women expectancy of life is reported. PRH - private health center; PLH - public health center

Age (years)	GP	PRH, no surgery	PLH, no surgery	PRH, surgery	PLH, surgery
50–59	30–35	3.8 (1.8–5.7)	3.3 (2.9–3.8)	21.0 (19.5–22.4)	12.5 (11.8–13.9)
60–69	21–26	3.0 (0–15)	2.1 (1.8–2.5)	12.5 (11.8–13.2)	8.1 (7.8–8.4)
70–79	14–18	1.9 (1.5–2.3)	1.4 (1.3–1.5)	7.4 (7.2–7.6)	5.1 (5.0–5.2)
>80	12–15	0.9 (0.7–1.1)	0.7 (0.7–0.8)	4.0 (4.0–4.1)	2.8 (2.8–2.9)

**Figure 3:**
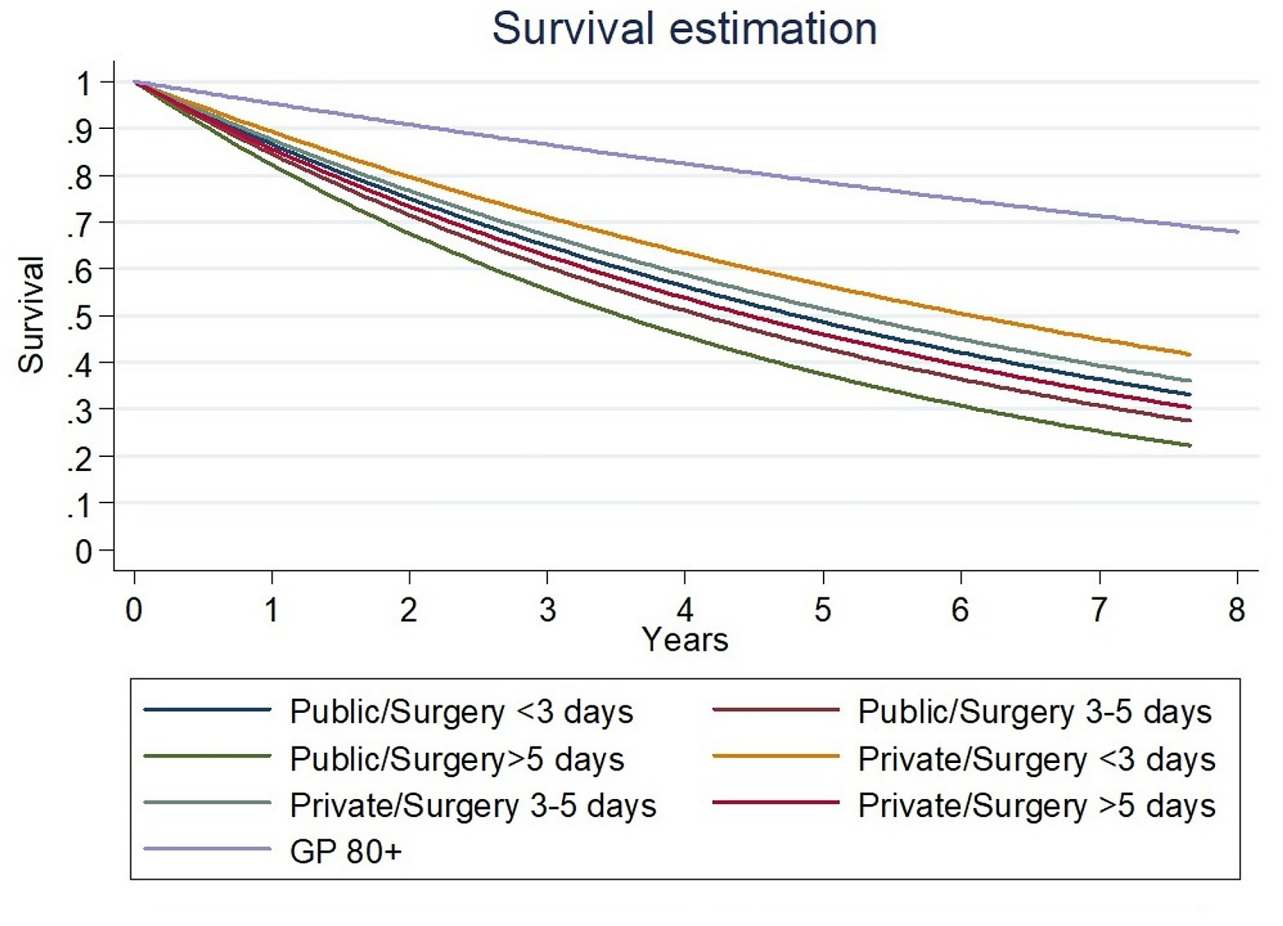
Survival curve estimated by exponential regression Survival curve estimated by exponential regression. From best to worse: general population over 80 years old (GP 80+), private center and surgery under three days, private center and surgery between three and five days, public center and surgery under three days, private center and surgery above five days, public center and surgery between three and five days, public center and surgery above five days.

The second exponential regression estimates an HR 1.05 (95% CI: 1.04 to 1.06) for each year in age, HR 1.61 (95% CI: 1.39 to 1.86) for men, HR 1.17 (95% CI: 1.07 to 1.26) for every day waiting for surgery, and an HR of 1.26 (95% CI: 1.05 to 1.53) for the PLH. This estimation shows that the waiting time for surgery has an impact on survival at any time. The estimated median survival time is summarized in Table 4. The survival curve estimated by the exponential regression model is shown in Figure 3.

**Table 4:** Life expectancy in years after hip fracture in patients that underwent surgery. Life expectancy in years and the 95% confidence interval-after hip fracture in patients that underwent surgery by type of health center, group of age, and waiting time for surgery (WTS). Also, the general national population (GP) is shown in the first row, on the left, the male expectancy of life and the right, the women expectancy of life is reported.

	Life expectancy (years)			
	50–59 years	60–69 years	70–79 years	>80 years
GP (male; female)	30; 35	21;26	14;18	12;15
WTS <3 days				
Private	20.7 (17.6–23.7)	11.8 (10.3–13.4)	7.7 (7.1–8.2)	4.3 (3.9–4.6)
Public	12.8 (11.5–14.0)	9.2 (8.5–9.9)	5.9 (5.6–6.1)	3.4 (3.2–3.5)
WTS 3–5 days				
Private	18.9 (11.9–26.0)	12.5 (11.6–13.5)	7.6 (7.1–8.1)	3.9 (3.7–4.1)
Public	14.4 (12.5–16.3)	8.3 (7.5–9.0)	5.6 (5.6–6.1)	3.2 (3.0–3.3)
WTS 5 days				
Private	13.8 (6.0–21.7)	8.8 (5.7–11.8)	5.8 (4.4–7.1)	3.5 (3.1–3.9)
Public	10.6 (9.8–11.4)	7.3 (6.9–7.6)	4.7 (4.5–4.8)	2.7 (2.6–2.8)

Median survival time after hip fracture is significantly higher in the PRH compared to the PLH and the cohort of Dinamarca-Montecinos et al. [10]. In addition, the one-year mortality rate is also significantly lower in the PRH group. Comparing the treatment in PLH to the cohort of Dinamarca-Montecinos et al. [10], PLH has a significantly higher median survival time and lower adjusted (AD) global one-year mortality (Table 5).

**Table 5:** Life expectancy and one–year mortality after hip fracture in patients over 50 years Life expectancy and one–year mortality after hip fracture in patients over 50 years. Life expectancy was estimated by exponential regression and adjusted by age and gender. One-year mortality is reported in three items. First is the summarized data. Second is the probability of dying one year after the hip fracture estimated with logistic regression and adjusted by age and gender. Last is the one–year mortality estimated after adjusting for the mean age and gender proportion reported in the study by Dinamarca-Montecinos et al. [10]. All data are accompanied by the 95% confidence interval. PRH - private health center; PLH - public health center

	PRH	PLH	Dinarmaca-Montecinos et al. (10)
Life expectancy (years)			
No Surgery	1.8 (1.2–2.5)	1.2 (1.1–1.4)	0.8
Surgery	6.9 (6.4–7.4)	4.5 (4.4–4.7)	3.0
Total	6.8 (6.3–7.29)	4.2 (4.1–4.4)	2.21
One-year mortality			
No surgery	0.27 (0.12–0.48)	0.65 (0.57–0.71)	0.58
Surgery	0.14 (0.12–0.16)	0.22 (0.20–0.24)	0.20
Total	0.16 (0.14–0.19)	0.26 (0.24–0.27)	0.27
Logistic regression			
No surgery	0.49 (0.41–0.58)	0.61 (0.54–0.68)	0.58
Surgery	0.14 (0.12–0.16)	0.21 (0.19–0.23)	0.20
Total	0.16 (0.13–0.18)	0.23 (0.21–0.25)	0.27
Adjusted by age and gender Dinamarca-Montecinos, et al.			
No surgery	0.49 (0.41–0.58)	0.61 (0.54–0.67)	0.58
Surgery	0.14 (0.12–0.16)	0.21 (0.19–0.23)	0.20
Total	0.16 (0.13–0.18)	0.23 (0.21–0.25)	0.27

## Discussion

Even though both centers have a one-year mortality rate comparable to international reports [12, 13], the most significant finding of this study is that, after hip fracture, patients over 50 years old treated in the PLH had a significantly higher one-year mortality and a lower life expectancy than those treated in a PRH. The two principal factors causing the difference are the proportion of patients who did not undergo surgery and the waiting time for surgery-each was significantly increased for the PLH patients.

A patient who does not undergo surgery after a hip fracture is at a higher risk of prostration or severe dependency increases, which increases the risk of aspirated pneumonia, skin ulcers, deep vein thromboembolism, pulmonary thromboembolism, depression, and death [14]. In Northern Ireland, only 1.3% of patients did not undergo surgery; the most frequent reasons for not undergoing surgery were high risk of mortality associated with surgery, or the patient died before surgery could be performed [15]. The Scottish guidelines for hip fracture treatment recommend patients should not receive surgery only in exceptional cases [16]. Accordingly, this study shows that patients who do not undergo surgery have a lower life expectancy and a higher mortality rate adjusted by age, gender, and center-type. One-year mortality in patients treated without surgery in PRH is 26%, which is very low compared to the PLH rate (65%). Interhospital transfers due to health insurance coverage that would allow surgery to become an option for a patient who may not otherwise receive it, may explain the low one-year mortality rate; this is a bias in the PRH cohort. Nevertheless, both centers must strive to reduce the conservative treatment rate, which is too high compared to the North Ireland cohort [15], especially in the PLH.

Waiting time for surgery has mainly been described as an essential risk factor for one-year mortality [17]. This study shows that delayed surgery also affects the expectancy of life after a hip fracture. The current recommendation of the Scottish guidelines for hip fracture treatment is that the patient should undergo surgery in less than three days [16]. Efforts should be made to reduce the waiting time of surgery in both centers as the PRH has 50% of the patients undergoing surgery after three days, while in the PLH group, 75% of patients undergo surgery in three days or more.

Operating room availability is crucial for reducing both the non-surgery rate and waiting time for surgery. Granting surgical priority to a patient with a hip fracture should not be a task for the orthopedic surgeon alone - it should concern all the medical and administrative staff of the health center [18]. The surgery should only be delayed if the patient requires another intervention for a condition that increases the risk of death over the risk of death from delaying the surgery [19]. The collaboration between the geriatrician, physician, orthopedic surgeon, and anesthesiologist will allow the surgery to be performed with a balance between minimum waiting time and comorbidity stabilization. A recent meta-analysis by Klestil et al. [20] shows that surgery within 48 hours was associated with lower mortality risk at one-year and fewer perioperative complications [19]. Also, Johanssen et al. [21] showed that the absolute perioperative mortality risk for hip surgery patients with an American Society of Anesthesiologists (ASA) score of four is lower than 1.5%.

In the Dinamarca-Montecinos et al. [10] cohort, 18% of patients did not undergo surgery, and only 8% of patients have a waiting time for surgery shorter than five days. These two factors seem to be the primary reasons for the significantly shorter life expectancy and adjusted one year-mortality compared to the PLH of this study. Two main reasons explain this difference. First, a special program was introduced in 2013 for fracture surgery that allowed for extra hours paid by performance, allowing for two additional fracture fixation operations per working day. This translated to an average of four more hip fracture fixation per week. Secondly, 2014 saw the inclusion of geriatricians in the preoperative and postoperative care of patients. The impact of these two factors is visible in the significant downward trending in-hospital length of stay, waiting time for surgery, and one-year mortality rate found in the PLH. Nevertheless, those efforts have not been enough to match the outcomes described in the PRH. Additionally, the significant difference found between PLH and the Dinamarca-Montecinos cohort [10], both belonging to the public network, raises a concern about inequality between different regions of Chile.

This study also shows that patients have a significantly shorter life expectancy adjusted by age and gender compared to the general population, despite the center and waiting time for surgery after hip fracture. This discovery should encourage additional efforts in prevention. Osteoporosis, sarcopenia, and falls are the major modifiable risk factors. Osteoporosis is currently one of the more significant epidemics and is often a misdiagnosis [22]. The implementation in primary care of a muscle training program in at-risk groups showed an improvement in balance and decreased the risk of falls [23, 24]. 

One of the more significant changes in Chile regarding public health strategies in the last 20 years has been the Explicit Health Guarantees (GES) - they constitute a set of benefits guaranteed by law allowing guaranteed access, opportunity, financial protection, and quality of care in a limited list of diseases. For example, one of the first pathologies included was acute myocardial infarction. After GES inclusion, one-year mortality significantly decreased (adjusted for age and gender) in the public health system [25]. Given the worldwide increase in the incidence of hip fracture and the impact of timely access to surgery, not only on mortality at one year but at the eight-year follow-up, this pathology is a serious candidate to be considered within the diseases listed in this health law. Additionally, its inclusion as a pathology in the law will allow systematic surveillance and auditing, which by itself had significantly improved hip fracture care in Scotland [26].

A limitation of this study is that other factors related to one-year mortality like ASA score, race, presurgery serum albumin levels, type of fracture, and adherence to the national program were not studied in this report [2, 27, 28]. The type of fracture, fixation method and ASA score was not analyzed, but as stated in the method section, during the period studied both centers had access to the same fixation method, and two senior hip surgeon worked in both centers, therefore, the selection criteria for the fixing method can be assumed to be homogeneous; moreover given the size of the sample and that all patients with a hip fracture were included (no selection bias) a normal distribution could be assumed in the type of fracture and ASA score, making the populations comparable. The sample size ensures that the distribution of the type of fracture and ASA score should probably be comparable. Also, social factors linked to the lack of social equity (such as social support and socioeconomic status) affects survival after hip fracture; nevertheless, this area lacks rigorously designed studies that measure the real impact on mortality after hip fracture [29]. Another limitation in this study is that the PRH data from 2002 to 2012 were manually registered; from 2012 onwards, an electronic file system was used, and this could be a source of bias. Also, the specific cause of mortality is not known (e.g., a patient could have a hip fracture but died because of cancer). Nevertheless, the number of patients studied minimizes this bias. Another limitation is the difference in volume between both centers. The PLH treated nearly twice as many patients in half the time compared to the PRH. However, mortality in hip fracture has not been associated with the patient load for an institution nor for the volume of the surgeon [30].

## Conclusions

Patients over 50 years old treated in PRH have a longer life expectancy and a lower probability of dying at one year after hip fracture compared with those treated in PLH. Waiting time to undergo surgery and duration of surgery are essential factors for the differences noted. Better access for hip fracture treatment is required in the public Chilean health system. This study is a strong call for attention to improve Chilean public health policies.
